# Leading teams while exhausted: Perspectives from healthcare epidemiology and beyond

**DOI:** 10.1017/ash.2022.25

**Published:** 2023-03-15

**Authors:** Rebecca A. Mullin, Susy S. Hota, Gonzalo Bearman

**Affiliations:** 1 Department of Internal Medicine, Virginia Commonwealth University Medical Center, Richmond, Virginia; 2 Infection Prevention and Control Department, University Health Network, Toronto, Canada; 3 Department of Medicine, University of Toronto, Toronto, Canada; 4 Division of Infectious Diseases, Virginia Commonwealth University, Richmond, Virginia; 5 Division of Infectious Diseases, Department of Internal Medicine, Virginia Commonwealth University Medical Center, Richmond, Virginia

## Abstract

Mental fatigue and burnout are concerns for healthcare organizations, but their effects on leaders have not been thoroughly studied. Infectious diseases teams and leaders are at risk for mental fatigue and burnout due to the increased demands from the coronavirus disease 2019 (COVID-19) pandemic, additive effects of severe acute respiratory coronavirus virus 2 (SARS-CoV-2) (omicron) and δ (delta) variant surges, and unique pre-existing pressures. No single intervention can reduce stress and burnout in healthcare workers. Work-hour limitations may have the biggest impact in physician burnout mitigation. Institutional and individual programs focused on mindfulness may improve well-being in the workplace. Leading during times of stress requires a multimodal approach and an understanding of goals and priorities. Greater awareness of burnout and fatigue across the healthcare spectrum and continued research are required to advance healthcare worker well-being.

*All organizations start with WHY, but only the great ones keep their WHY clear year after year.*

Simon Sinek, *Start with Why*

Missed steps, mistakes, numbness to critical stimuli, and irrational decision making are all potential consequences of fatigue. When a healthcare worker experiences mental fatigue, results can be harmful to patients, self, and others. Consequently, close attention is focused on preventing and treating burnout and mental fatigue in frontline healthcare workers. But the effects of mental fatigue experienced by those in healthcare leadership positions has not been studied as thoroughly.

Mental fatigue in leaders results in more insidious consequences, undermining teams and subverting efforts to provide safer, more efficient health care delivery. Even before the coronavirus disease 2019 (COVID-19) pandemic, a Medical Group Management Association (MGMA) survey of US healthcare leaders indicated that >60% experienced some degree of burnout.^1^ The severe acute respiratory coronavirus virus 2 (SARS-CoV-2) (omicron) and δ (delta) variants led to further surges in an already stressed healthcare system. A recent article in *The Atlantic* described variant surges as an “additive burden,” citing high numbers of healthcare employee resignations and COVID-19 infections resulting in a thinning workforce left to care for patients admitted to hospitals.^2^ Although efforts to measure and define mental fatigue grow, research specific to mental fatigue in leaders, and the effects on healthcare teams, remains comparatively sparse. Individuals working in the fields of infectious diseases, epidemiology, and public health are called upon to provide leadership within healthcare organizations both through formal administrative positions and informal expert guidance. Without stronger evidence-based strategies for management of mental fatigue, infectious diseases leaders face will continue facing challenges in self-care and prevention of burnout in teams.

## Mounting pressure on infectious diseases experts

The COVID-19 pandemic placed new reliance on the expertise of healthcare infectious diseases teams to develop pandemic response guidance, to prevent outbreaks, and to help manage the flow of patients and providers in healthcare facilities. Simultaneously, in a recent article “Virtual Infection Prevention—The Next Frontier,” K. Barrows pointed out, “Healthcare trends in physician compensation, market consolidation and private equity have contributed to [a] shortage of infectious diseases specialty support.”^3,4^ Additionally, the Association of American Medical Colleges projects a shortage of 3,800–13,400 physicians in non–primary-care medical specialties by 2034, while the US population ages and increases demand.^5^ Staffing levels correspond to mental fatigue in nursing teams, and a recent meta-analysis reported that frontline healthcare workers are at higher risk for mental health issues related to the global crisis including posttraumatic stress disorder, anxiety, depression, and sleep disorders.^6^ Efforts to bolster the infectious diseases workforce have resulted in incremental gains. For example, the 2021 US match rate for open infectious diseases fellowships was 75%, up from only 42% in 2016. However, attention must be given to staffing shortages and potential impacts on mental fatigue in the workforce.^6^ No comparable data are available to elucidate the relationship between epidemiology and infectious diseases services staffing levels and burnout or mental fatigue.

## Potential unique drivers of burnout in healthcare epidemiology

No published reports exist on the unique drivers of burnout in healthcare epidemiology. Healthcare epidemiology is unique given its focus on population medicine, implementation, and healthcare administration.^7^ Physician burnout in healthcare epidemiology may result from challenges in influencing system and population-level practice change.^7^ Other concerns include potential health-system value misalignments through understaffing, under-resourcing of safety programs, and perceived lack of respect and support from hospital executives and physician colleagues.^7^ These stressors are compounded by pressures of the COVID-19 pandemic.

## Individual level factors for leading while exhausted

For all leaders, the critical step to any important decision is to understand higher-level goals. What is the underlying purpose and what impact will it have on ourselves and others? With that, leaders must effectively clarify what is urgent versus nonurgent yet important. This fosters prioritization and may decrease deadline stress.

A key individual strategy is to balance compassion and containment so that one may care personally and provide direct team feedback.^8^ Under this framework, leaders recognize team members for who they are and not what they do. Listening to team members regularly is necessary for course-adjusting strategies. Leaders must also feel comfortable functioning in uncertainty and expressing “I don’t know.” Promoting stability during chaos requires setting limits for teams, defining clear expectations, keeping pressure at optimal levels, minimizing tasks appropriately, including reducing the time in frequency of meetings, reducing, or cutting unnecessary projects, concisely communicating, and minimizing excessive e-mails.

A critical re-examination of personal and team resilience is required to accurately gauge the capacity and strength of the group. Strategies to foster resilience include cultivating compassion, taking deliberate and timed detachment breaks, developing mental agility through pause and observation for better decision making and greater flexibility as well as exercising mindfulness.^9^ Importantly, even the best leaders make mistakes, lose focus, and choose poorly. What matters is how you recover from mistakes—through honesty, personal responsibility, reflection, and positive action, with a growth mindset.

## Focus on team-level dynamics

As the pandemic continues, healthcare epidemiologists and healthcare leaders must be cognizant of multimodal approaches to minimize the negative impact of stress on a team. This includes recognizing the symptoms and consequences of individual and team fatigue. Although no perfect working environment exists, a recent report suggests that employee performance (productivity) optimization is not enough, especially when the bulk of modern work is team based.^10^

In a study of >180 teams at Google, researchers concluded that the highest functioning teams were not a reflection of the ‘who’ or make-up of the work unit; team norms were most important.^10^ The highest functioning teams were characterized by psychological safe environments (norm) leading to team binding, where leaders encourage and promote honest and compassionate conversations about ideas, challenges, frictions and everyday annoyances to meet the needs of the team and the goals of the enterprise.^10^ Leaders should advocate to the fullest extent possible for ongoing professional tasks and projects that continue to provide meaning and professional satisfaction to team members, such that work is addressed with purpose, autonomy and mastery. Team dynamics must be continually assessed and adjusted such that the group function effectively with work that is purposeful, personally integrated, and not just focused on efficiency.

## System level changes to minimize burnout

No single intervention on a system level effectively reduces healthcare worker burnout. In a meta-analysis of 15 randomized controlled trials and 37 cohort studies that included interventions such as, mindfulness training, stress management, small group discussions and duty hour reductions/workload reductions, researchers concluded that individual and organizational interventions may reduce physician burnout by 10% (from 54% to 44%).^11^ Organizational efforts such as work-hour limitations, workflow improvements, and biweekly discussion groups with mindfulness, reflection, shared experiences and small-group learning result in modest yet significant reductions in Maslach Burnout Inventory surveys in healthcare workers.^12^ Key organizational bundled strategies to reduce burnout include acknowledging and assessing the problem, harnessing the power of leadership, developing and implementing targeted work unit interventions, cultivating a community of work, using awards and incentives, aligning practice and values, promoting flexibility and work life integration, and providing research to promote resistance and self-care.^13^ Evidence suggests that physicians who spent at least 20% of the professional effort focused on the dimension of work they find most meaningful are significant at a lower risk for burnout.^13^

Ultimately, systemwide changes are required at the level of the organization so work hours are not excessive, workflow is optimized to minimize unnecessary tasks, support groups are available, and the regular measurement of burnout and fatigue is part of a culture of safety.
